# Editorial for the Special Issue “Molecular Bases of Senescence”

**DOI:** 10.3390/ijms222111873

**Published:** 2021-11-02

**Authors:** Giorgio Fanò-Illic, Stefania Fulle, Patrizia Mecocci

**Affiliations:** 1 IIM-Interuniversity Institute of Myology, University “G. d’Annunzio” of Chieti-Pescara, 66100 Chieti, Italy; stefania.fulle@unich.it; 2 Campus of Free University of Alcatraz, Santa Cristina di Gubbio, 06100 Perugia, Italy; 3 A&C M-C Foundation for Translational Myology, 35100 Padova, Italy; 4 Department of Neuroscience Imaging and Clinical Sciences, University “G. d’Annunzio” of Chieti-Pescara, 66100 Chieti, Italy; 5 Institute of Gerontology and Geriatrics, Department of Medicine and Surgery, University of Perugia-Santa Maria della Misericordia Hospital, 06100 Perugia, Italy; patrizia.mecocci@unipg.it

The increasing life expectancy of populations worldwide represents the most evident success of the last century thanks to varying interacting social and medical achievements.

However, the oldest age period of life is still characterized by long-lasting disability, showing how the success in increasing age has not been followed by the maintenance of good health status. In fact, many chronic diseases cannot yet be entirely prevented, and this makes it difficult to guarantee a satisfying quality of life.

As such, it is evident that biological and medical sciences now must face new challenges due to the role of aging as the leading risk factor of age-related and chronic diseases—including neurodegenerative disorders, sarcopenia, osteoporosis, etc.—with a rising number of affected subjects. Understanding the role of the molecular alterations causing cellular senescence may offer new opportunities to reduce the impact of aging on individual health status and define preventive and therapeutical strategies capable of reducing the risk of suffering from age-related chronic diseases. Furthermore, several studies are now focusing on the detection of biomarkers that can reveal the presence of an altered status before the clinical expression of a specific disease, and this field of research is a promising area for increasing our capacity to identify subjects at risk and offer more efficacious approaches.

Alzheimer’s disease (AD) is one of the most studied pathologies for detecting biomarkers capable of revealing preclinical stages of disease and the efficacy of specific treatments. A paper by Casoli and coll. [1] has focused on the role of the platelet APP ratio (i.e., the ratio between the two different isoforms of the amyloid precursor protein, APP, involved in central and peripheral synaptogenesis) as a peripheral measure of synaptic plasticity. In particular, they evaluated the effect of a cognitive training program in a group of subjects with AD compared to an untreated group. Although a positive effect of cognitive training was evident in the short run, with a loss of efficacy after two years, the statistically significant relationship between the platelet APP ratio and several cognitive functions in the non-treated group was of interest. This aspect is controversial, since more active neuroplasticity was expected in the active group but could also reflect a reduced production of APP, which is a precursor of amyloid peptides in AD. Further studies might better clarify the significance of the platelet APP ratio as a biomarker in AD detection and progression.

The possibility of modulating the effect of senescence in the brain has been studied by Sola-Sevilla and coll. [2]. They evaluated the impact of overexpression of isoform 3 of the protein sirtuin 2 (SIRT2.3) in the hippocampus of senescence-accelerated mouse-prone 8 (SAMP8) mice compared to controls. SIRT is a family of seven different enzymes (SIRT 1–7) capable of epigenetically modulating DNA expression through histone deacetylation; they are essential factors in delaying cellular senescence and extending the organismal lifespan. SIRT2 is located in the cytoplasm and is able to move to the nucleus and mitochondria, having a large number of modulated substrates. As such, it is involved in several functions, with strongest expression in the brain. Although an accumulation of SIRT2.3 has been reported in the aging brain, this study did not find a protective effect of its overexpression in either SAMP8 or control mice, both from a molecular and a behavioral point of view. However, in SAMP8, the increased amount of SIRT2.3 was associated with higher expression of the inflammatory protein Il-1b. This leaves open the hypothesis of an interaction between different factors in aging, with the activation of molecular pathways having a final detrimental effect. In fact, SIRT2 inhibitors reduce neuroinflammation, which is involved in the pathogenesis of age-related diseases such as AD.

Even in ageing kidneys, which are characterised by increased vulnerability to glomerulosclerosis and a measurable decline in kidney function, enzymes of the sirtuin family (in particular SIRT-1) play an essential role. Evidence suggests that renal and systemic deficiencies of klotho (which appears to prolong lifespan, but whose level decreases with age) and SIRT1 worsen renal damage induced by exogenous stress. The aim of the study presented by Chen et al. [3] was to explore whether resveratrol could attenuate renal oxidative stress induced by concanavalin A in elderly mice. The results obtained demonstrated that plasma and urinary levels of renal damage markers were significantly increased in aged mice treated with con A. In addition, kidneys of aged mice also showed markedly increased levels of reactive oxygen species and DNA damage, with reduced levels of glutathione, klotho and SIRT1 after con A treatment. These results suggest that resveratrol protects against con A-induced advanced glomerulosclerosis in aged mice by ameliorating renal oxidative stress through SIRT1-mediated klotho expression (Figure 1).

It is well documented that ageing also reduces the barrier function and turnover of the intestinal epithelium, resulting in increased systemic inflammation. In Lee et al.’s [4] study, age-associated changes in the gut were examined by microRNA profiling in isolated mouse intestinal epithelial cells. The results of the research confirmed this hypothesis by showing that in relation to ageing, there is dysregulation of different miRNA species, with a greater prevalence in small epithelial cells (22 miRNAs) than in large cells (3 miRNAs).

However, senescent cells secrete pro-inflammatory factors through a secretory phenotype associated with senescence that is very particular to this cellular situation. In the paper of Song and Bae [5], the effects of protein kinase CK2 on the expression of some of the factors involved in the senescence-associated secretory phenotype, including interleukin IL-1β, IL-6, and MMP3, were investigated. The data presented here indicate that during the aging process, an enhancement of the senescence-associated secretory phenotype (SASP) occurs through down-regulation of CK2 via dysregulation of NF-κB (IκB) transcription factors and the AKT-IκB kinase axis. Interestingly, the AKT inhibitor triciribine and the SIRT activator resveratrol significantly abrogated the increased expression of the genes involved.

The importance of the SASP is also demonstrated in a mutation in the ATP-binding cassette sub-family C member 6 gene leading to the appearance of pseudoxanthoma elasticum (PXE), which is a syndrome clinically characterised by a loss of skin elasticity, and atherosclerosis, associated with molecular aspects typical of premature ageing. Using primary cultures of human dermal fibroblasts, Tiemann et al. [6] highlighted a potential connection between premature cellular ageing and the pathogenesis of PXE, highlighting the correlation between peculiar aspects of the senescent phenotype and certain factors involved in cell cycle control—in particular, an increase in β-galactosidase activity associated with senescence as well as an increase in the expression of pro-inflammatory factors such as IL-6 and MCP1. An increase in gene expression of the cyclin-dependent kinase inhibitor (CDKI) p21 is also shown, but no simultaneous induction of p53 gene expression. A permanent DNA double-strand break in response to ionising radiation triggers a prolonged response to induced damage of nucleic acids and premature senescence. The resulting deep chromatin reorganisation begins with the formation of heterochromatin foci, an essential mechanism for controlling SASP. To decipher the molecular mechanisms underlying this process, both chromatin remodelling and double-strand damage through the dynamics of histone H2A.J variant incorporation were analysed in human fibroblasts after exposure. The results presented in the paper by Isermann et al. [7] provide mechanistic insights into the biological phenomena of SASP and suggest that inhibition of H2A.J might even eliminate it.

In the skeletal muscle, ageing causes a decline in function with progressive loss of muscle mass, quality and strength (sarcopenia). Along with this functionally impaired situation, the regenerative system also shows a dramatic reduction. A key role in maintaining proper functional status is played by the extracellular matrix (ECM) that supports tissue structure in skeletal muscle. As is well known, gene expression of many components of the ECM decreases with age, leading to an accumulation of collagen in skeletal muscle. In the paper by Chen et al. [8] it seems well established that, with advancing age, ECM degradation undergoes negative remodelling due to the downregulation of several genes such as TGF-β, some tissue inhibitors of metalloproteinases (TIMPs), matrix metalloproteinases (MMPs) and cathepsins. If muscle injury can induce muscle repair and regeneration in young skeletal muscle, this could effectively induce regeneration in aged skeletal muscle. This hypothesis is interesting and would also be easy to test in practice, but the available data are still preliminary.

From a mechanical properties point of view, senescent muscle shows an increase in stiffness, but it is still debated whether it is the muscle fibres or ECM that are responsible for this change. To answer this question, Pavan et al. [9] compared the passive stress generated by stretching fibres individually and when arranged in small bundles in young healthy subjects (average age 21 years) and elderly subjects (average age 67 years). Both the physiological sarcomere length range and the area of ECM between muscle fibres were determined using a specific staining for collagen fibres. The passive tension of the fibre bundles was significantly higher in the elderly than in the young subjects, but the elongation resistance of the fibres alone was not different between the two groups. The contribution of ECM was also significantly increased in the elderly. In conclusion, age-related reduced compliance in human skeletal muscle is due to increased stiffness of the ECM, caused primarily by collagen accumulation.

Exercise has enabled skeletal muscle to counteract age-related sarcopenia by inducing a wide range of adaptations, supported by the expression of genes encoding proteins involved in energy management, proteostasis, cytoskeletal organisation, control of inflammation and cellular senescence. De Sanctis et al. [10] had previously examined the entire transcriptome of the vastus lateralis muscle of sedentary elderly and similarly aged athletes with an exceptional record of high-intensity, lifelong training. In this paper, the authors describe the network of differentially expressed non-coding RNAs in the two classes and some of their possible targets and roles (Figure 2). 

Hierarchical clustering analyses of all non-coding RNAs were able to discriminate between sedentary and trained individuals, regardless of exercise type. Validated targets of differentially expressed miRNAs were clustered by KEGG analysis, which indicated that the functional areas involved are cytoskeletal control, longevity and several signalling pathways (including AMPK and mTOR), which have been shown to be crucial in modulating the effects of high-intensity, lifelong training. Analysis of differentially expressed RNAs also identified transcriptional networks involving lncRNA, miRNA and mRNA, that influence processes consistent with the beneficial role of exercise training. In addition, in this Issue, the same group (see Bolotta et al. [11]) demonstrated that unsupervised mixture distribution analysis was able to correctly classify trained and untrained subjects, while it failed to discriminate between individuals who underwent long-term resistance training (*n* = 5) or long-term endurance training (*n* = 4), thus demonstrating that the training mode was not relevant for the prevention of sarcopenia.

Finally, two reviews have highlighted two particular aspects that are linked to the senescence of organs and apparatuses: the regenerative capacity of staminal cells and the proteic regulation of multi-organ processes.

The use of mesenchymal stromal cells (MSCs) in regenerative medicine and tissue engineering is well established, given their self-renewal and differentiation properties. However, several studies have shown that these properties diminish with age. In a PRISMA systematic review, Kapetanos et al. [12] described the effects of chronological ageing of donors on MSC senescence. A total of 3023 studies were derived from four databases: PubMed, Web of Science, Cochrane and Medline. Only nine studies met the inclusion and exclusion criteria and were included in the final analyses. These studies showed an increase in the expression of p21, p53, p16, ROS and NF-κB with chronological age. This implies an activation of the DNA damage response as well as increased levels of stress and inflammation in MSCs from older donors. In addition, a decrease in the expression of proliferative markers including Ki67, MAPK pathway elements and Wnt/β-catenin pathway elements was observed. Finally, an increase in the levels of SA-β-galactosidase, a specific marker of cellular senescence, was highlighted. Taken together, these results support an association between chronological age and MSC senescence, although the precise threshold of chronological age at which the reported changes become significant has yet to be defined and should form the basis for further investigation.

Over the last decade, clear evidence has emerged that the cellular components of skeletal muscle are important sites for the release of proteins and peptides called ‘myokines’, suggesting that skeletal muscle plays the role of a secretory organ. In summary, myokines influence complex multi-organ processes, such as skeletal muscle trophism, metabolism, angiogenesis and immunological response to different physiological (physical activity, ageing, etc.) or pathological (cachexia, dysmetabolic conditions, chronic inflammation, etc.) states (Figure 3).

The aim of the review by Mancinelli et al. [13] is to describe in detail a number of myokines that are, to varying degrees, involved in skeletal muscle ageing processes and that belong to the group of proteins present in the functional environment surrounding the muscle cell known as the ‘Niche’. The particular myokines described are those that, acting both from within the cell and in an autocrine manner, have a defined relationship with the modulation of oxidative stress in muscle cells (mature or stem) involved in the regulatory (metabolic or regenerative) processes of muscle ageing. Myostatin, IGF-1, NGF, S100 and irisin are examples of specific myokines that have peculiar features in their mechanisms of action. In particular, the potential role of one of the most recently characterised myokines, irisin, is discussed in terms of its possible application as an agent to counteract the deleterious effects of muscle ageing.

## Figures and Tables

**Figure 1:**
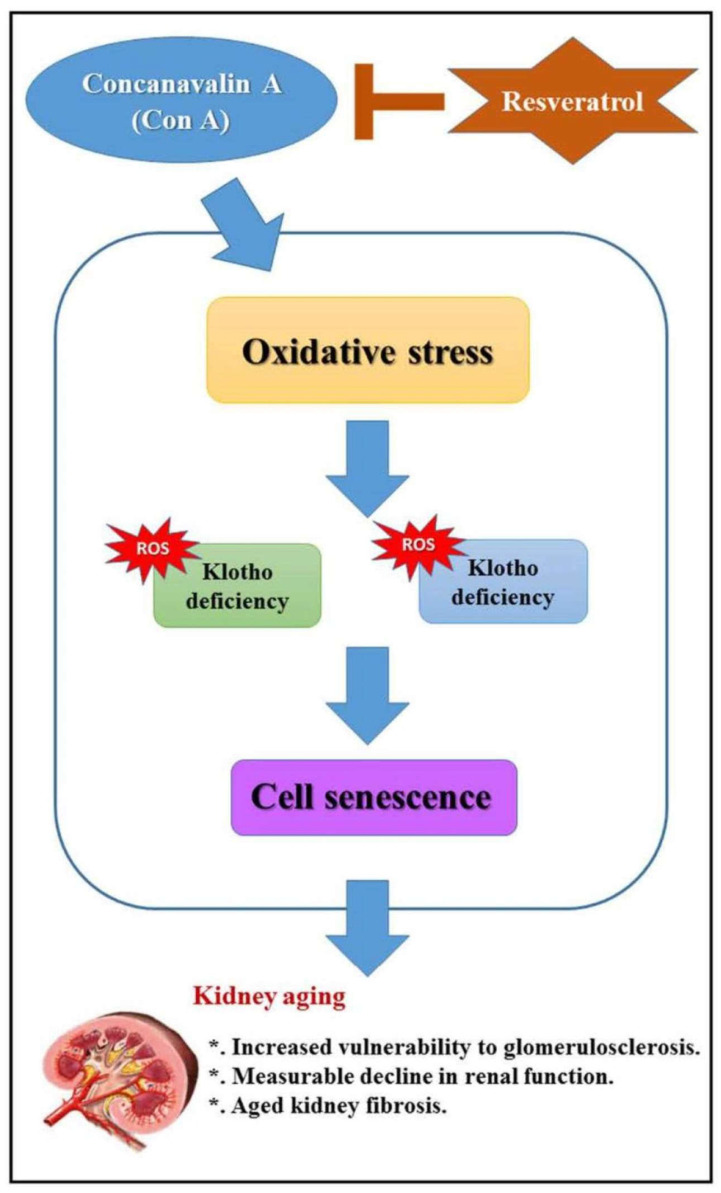
Schematic diagram of resveratrol pretreatment to prevent against concanavalin A (Con A)-induced advanced renal oxidative stress and glomerulosclerosis in aged mice. From Chen, C.-C.; Chang, Z.-Y.; Tsai, F.-J.; Chen, S.-Y. *Int. J. Mol. Sci.* **2020**, *21*, 6766. https://doi.org/10.3390/ijms21186766 (this Special Isue, [3]).

**Figure 2:**
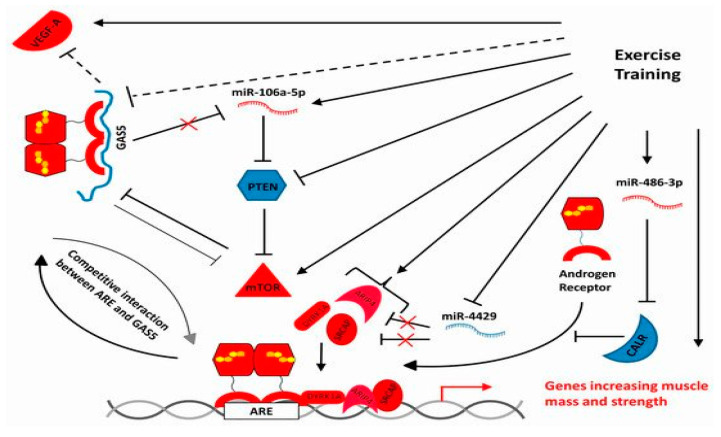
A simplified transcriptional network illustrating hypothesized interactions involving one lncRNA, three miRNAs and some protein-coding genes in the vastus lateralis of senior sportmen [10]. From De Sanctis, P.; Filardo, G.; Abruzzo, et al. *Int. J. Mol. Sci.* **2021**, *22*, 1539. https://doi.org/10.3390/ijms22041539 (this Special Isue, [10]).

**Figure 3:**
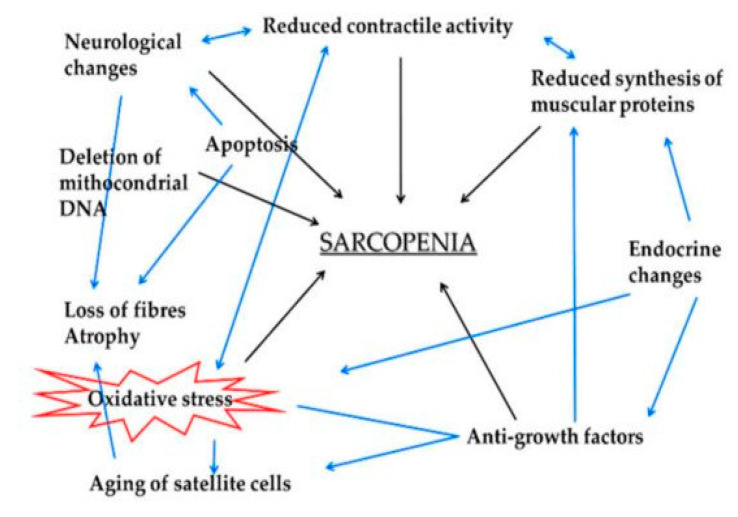
Skeletal muscle aging is a complex process that is associated with a decrease in mass, strength and velocity of contraction, known as sarcopenia. From Mancinelli, R.; Checcaglini, F.; Coscia, F.; et al. *Int. J. Mol. Sci.* **2021**, *22*, 8520. https://doi.org/10.3390/ijms22168520 (this Special Isue, [13]).
